# Statement of retraction: suppression of lncRNA GAS6-AS2 alleviates sepsis-related acute kidney injury through regulating the miR-136-5p/OXSR1 axis in vitro and in vivo

**DOI:** 10.1080/0886022X.2026.2660890

**Published:** 2026-04-23

We, the Editor and Publisher of *Renal Failure* have retracted the following article:

**Cui, H., Ren, G., Hu, X., Xu, B., Li, Y., Niu, Z., & Mu, L. (2022). Suppression of lncRNA GAS6-AS2 alleviates sepsis-related acute kidney injury through regulating the miR-136-5p/OXSR1 axis** *in vitro* **and** *in vivo***. Renal Failure, 44(1), 1071–1083.** https://doi.org/10.1080/0886022X.2022.2092001

After publication, concerns were raised by a third party in 2024 regarding the reuse of Figures 1D and 5 F from previously published articles of unrelated author groups.

Further investigation conducted by the Journal and Publisher identified significant integrity concerns, including the following:

- Unexpected overlaps in Figure 5F of the article with the immunohistochemistry images in the following previous publication from an unrelated author group:
  Huiping Zhu, Sen Zhong, Haihong Yan, Kangyao Wang, Lingwei Chen, Min Zhou, Yunsheng Li. Resveratrol reverts Streptozotocin-induced diabetic nephropathy. *Front. Biosci. (Landmark Ed)* 2020, 25(4), 699–709. https://doi.org/10.2741/4829
- Unexpected overlaps in Figure 3D and the Western blots of the following previous publication from an unrelated author group:
  Bing Z-X, Zhang J-Q, Wang G-G, Wang Y-Q, Wang T-G, Li D-Q. Silencing of circ_0000517 suppresses proliferation, glycolysis, and glutamine decomposition of non-small cell lung cancer by modulating miR-330-5p/YY1 signal pathway. Kaohsiung J Med Sci. 2021;37:1027–1037. https://doi.org/10.1002/kjm2.12440
- Additional integrity concerns regarding the Western blot data were identified due to similarities within the Western blots in the article.

When approached for an explanation, the authors did not respond to the queries raised by the Journal and Publisher.

As verifying the validity of published work is core to the integrity of the scholarly record, we are therefore retracting the article. The corresponding author has been informed.

We have been informed in our decision-making by our editorial policies and the COPE guidelines.

The retracted articles will remain online to maintain the scholarly record, but they will be digitally watermarked on each page as ‘Retracted’.

